# Supplementary material for: Chiral high-harmonic generation and spectroscopy on solid surfaces using polarization-tailored strong fields
Source: Nat Commun. 2021 Jun 17;12:3723. doi: 10.1038/s41467-021-23999-9 (PMC8211651; doi:10.1038/s41467-021-23999-9)
Supplement: Supplementary file 1 — Supplementary Information [file 41467_2021_23999_MOESM1_ESM.pdf]

Chiral high-harmonic generation and spectroscopy on solid surfaces using polarization-tailored strong fields

Tobias Heinrich[1], Marco Taucer[2], Ofer Kfir[1,3], P. B. Corkum[2], André Staudte[2], Claus Ropers[1,3], Murat Sivis[1,3]*

[1]4th Physical Institute – Solids and Nanostructures, University of Göttingen, 37077 Göttingen, Germany.

[2]Joint Attosecond Science Laboratory, National Research Council of Canada and University of Ottawa, 100 Sussex Drive, Ottawa, Ontario K1A 0R6, Canada.

[3]Max Planck Institute for Biophysical Chemistry, 37077 Göttingen, Germany.

*Correspondence to: murat.sivis@uni-goettingen.de.

Supplementary information

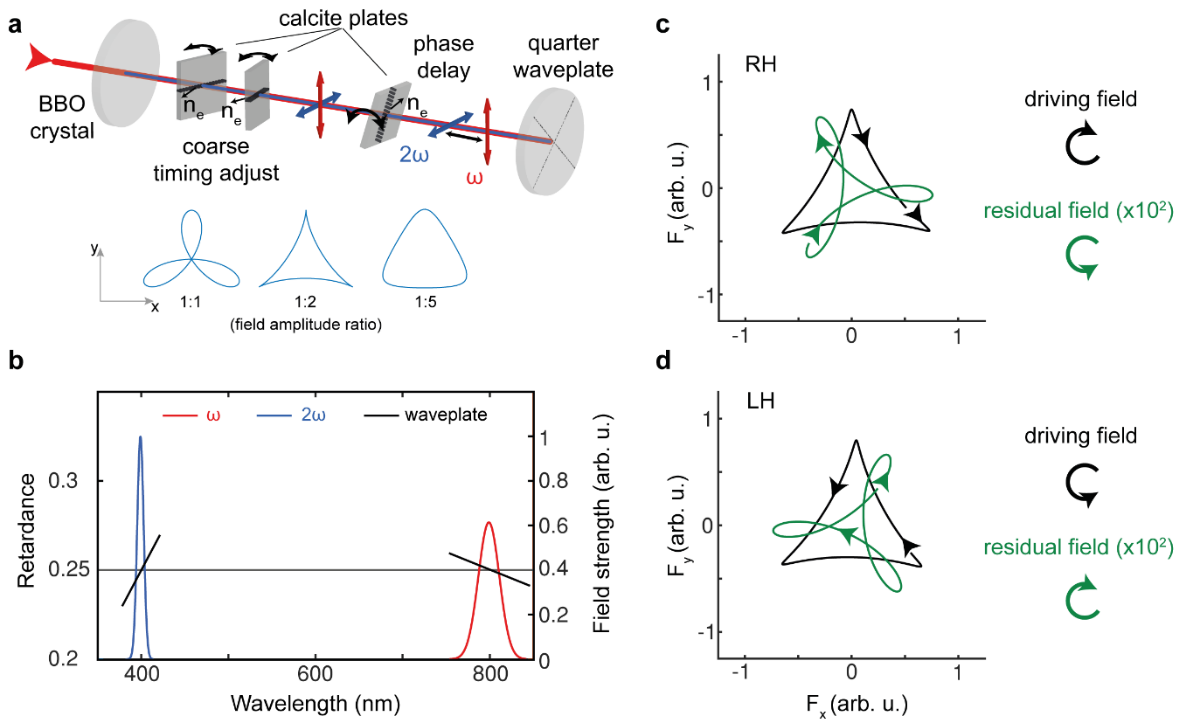

Supplementary Figure S1 | Three-fold bi-chromatic driving field. a, Schematic representation of the MAZEL-TOV apparatus used for generation of the bi-chromatic three-fold driving field. The timing between fundamental and second harmonic is adjusted to zero by a pair of calcite plates. An additional calcite plate is used to fine tune the phase and allow tunability of the cloverleaf orientation. Inset: Lissajous curves of driving fields for different field amplitude ratios (F_ω:$F_{2\omega}$) of the fundamental and second harmonic. **b**, Shown are gaussian fits of the measured incident spectrum (red and blue) and the specified retardance of the quarter-wave plate (black). **c**,**d**, Lissajous curve of the total three-fold right-helical (RH) and left-helical (LH) driving field (black) and the residual

field (green) for an amplitude ratio of 1:2. ne; optical axis, ω; fundamental frequency, 2ω; second harmonic frequency, $F_{x,y}$; electric field components.

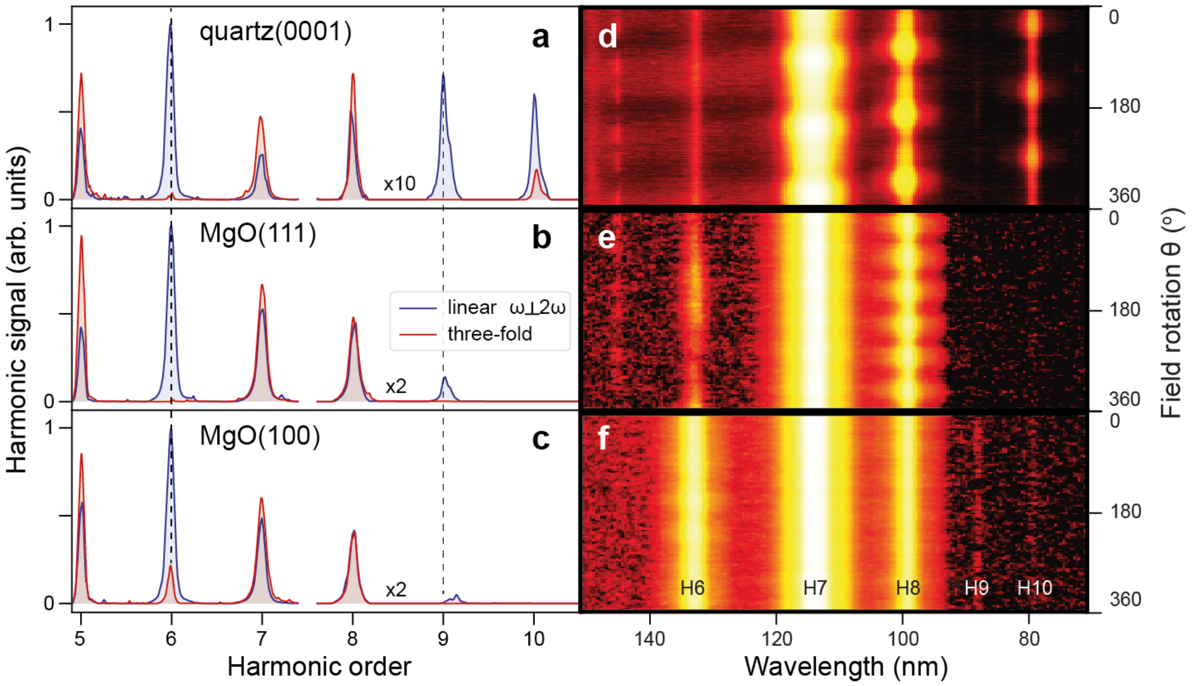

Supplementary Figure S2 | High-harmonic spectra. (**a**) quartz(0001), (**b**) MgO(111) and (**c**) MgO(100) spectra for two-color excitation with perpendicular-linear (blue) and bi-circular (red) polarization. Spectrograms for (**d**) quartz(0001), (**e**) MgO(111) and (**f**) MgO(100) as a function of three-fold field rotation angle θ with logarithmic color scale. The 8th harmonic of the spectrograms is used to generate Figs. 2a-c.

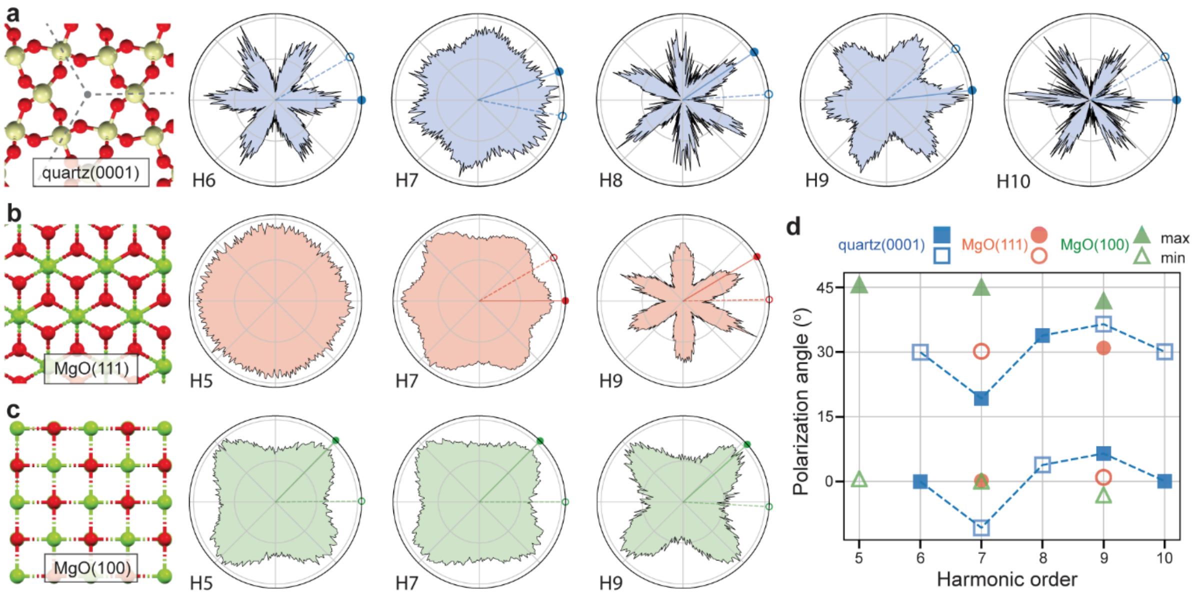

Supplementary Figure S3 | Linear Polarization Scans. Harmonic yields as a function of angle of linear polarization with respect to the fixed crystal, for (**a**) quartz(0001), (**b**) MgO(111), and (**c**) MgO(100), and their crystal structures. Dashed lines and filled/empty circles indicate angles corresponding to maxima/minima of the harmonic signal. **d**, Polarization angles corresponding to maximum and minimum signal for each material as a function of harmonic order (filled and empty shapes, respectively).

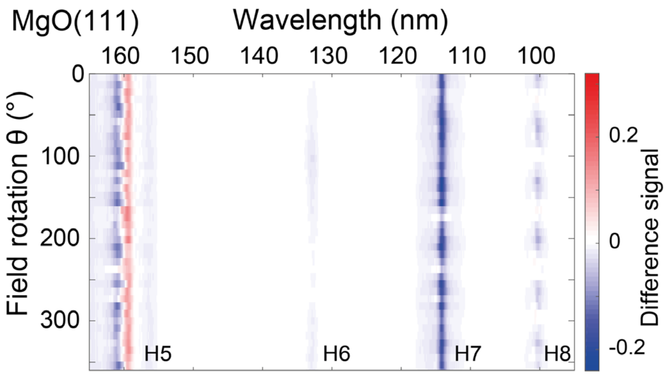

Supplementary Figure S4 | Field rotation scans on MgO(111). Difference signal from two intensity-normalized phase scans, recorded with left- and right-helical bi-circular driving fields, respectively.

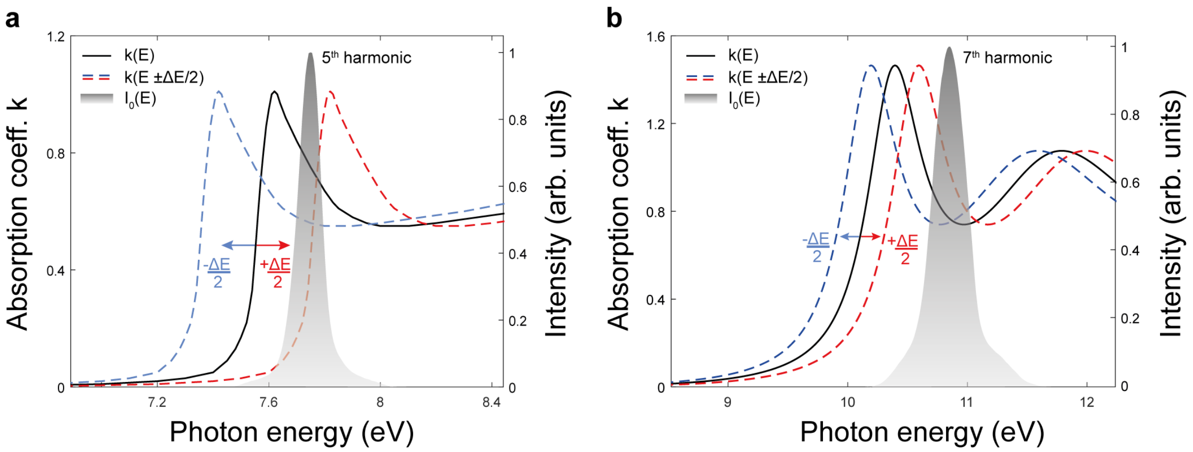

Supplementary Figure S5 | Model of shifted absorption edges. The imaginary part of the refractive index $k(E)$ (black line) for (**a**) MgO and (**b**) quartz. An energetically up (red) and down (blue) shifted edges model the effective absorption for left and right circular harmonics. The intensities $I_0(E)$ of the 5th and 7th harmonics are indicated

Selection rules for bi-circularly polarized HHG in solids

Harmonic generation is a multi-photon process which can involve many photons of each light field. Since the emitted photon can only have ±1 unit of angular momentum, and the two-color light fields used in this study have opposite circular polarizations, the possible combinations are constrained. In the case of an anisotropic solid, the lattice itself participates in the process and can give or take angular momentum on a femtosecond timescale, but only in quantized units given by its rotational symmetry. Allowed processes for harmonic generation must obey

$$q - 3n = 2lN \pm 2,$$

where q is the order of the emitted harmonic, n is the number of ω-photons, N refers to the rotational symmetry of the N-fold crystal, l is the number of quanta of angular momentum contributed by the lattice, and the sign determines the polarization state of the harmonic. This

selection rule is substantially different from the one derived for single color circular fields[1,2]. By energy conservation, the number of 2ω-photons must be $m = (q - n)/2$. Elementary algebra shows that when N and q are both multiples of three, there exist no solutions. That is, three- and six-fold materials forbid the production of harmonics 3, 6, 9, 12, etc. The relevant rotational symmetries in this case refer to rotation about the propagation axis of the light. This yields an explanation for the presence or absence of a selection rule depending upon the crystal orientation in the case of MgO. Figure S2 shows the high harmonic spectra emitted from the quartz(0001), MgO(111) and MgO(100) surface for bi-circular (red) polarization. Spectra recorded with perpendicular polarized linear pulses (blue) serve as a reference experiment. The high harmonic intensity generated by the bi-circular driving field shows near-perfect suppression of H6 and H9 for quartz(0001) and MgO(111). In the case of MgO(100) a much less pronounced suppression indicates a lifting of the selection rule by the four-fold rotational symmetry.

Linear polarization scans

For each crystal, we also performed angle-dependent measurements with linearly-polarized, single-color driving fields. As the 800 nm driving polarization is rotated with respect to the fixed crystals, we see variations in the harmonic yields (Fig. S3 and 2d-f). Maxima (minima) for each harmonic are indicated by a solid line and a filled (empty) circle. In these linear polarization scans, quartz(0001) and MgO(111) show a six-fold response, while MgO(100) appears four-fold, consistent with the respective three-fold, six-fold and four-fold crystal symmetries. For MgO, figure S5b and c, the maxima and minima are always along high-symmetry directions, although in the case of MgO(111) there is a reversal between harmonics 7 and 9. On the other hand, quartz(0001) shows a more complex angle-dependence, with each harmonic maximized along a different direction, as shown in figure S3d. The relative phase of the harmonics has been intensively studied and applied in various studies[3-7]. In a real space picture, the phase is determined by the electron trajectory of the individual harmonics. The more complex angle-dependence of quartz can likely be attributed to its more complex crystal structure. For example, the projections of chemical bonds onto the surface, evident in the left panels of figure S3a-c, are at several non-trivial angles for quartz, compared to the relatively simple bonding character of MgO.

References

1. Tang, C. L. & Rabin, H. Selection Rules for Circularly Polarized Waves in Nonlinear Optics. *Phys. Rev. B*. **3**, 4025–4034 (1971).
2. Saito, N. *et al.* Observation of selection rules for circularly polarized fields in high-harmonic generation from a crystalline solid. *Optica*. **4**, 1333–1336 (2017).
3. Luu, T. T. & Wörner, H. J. Observing broken inversion symmetry in solids using two-color high-order harmonic spectroscopy. Phys. Rev. A 98, 041802 (2018).
4. Luu, T. T. & Wörner, H. J. High-order harmonic generation in solids: A unifying approach. *Phys. Rev. B* **94**, 115164 (2016).
5. Luu, T. T. & Wörner, H. J. Measurement of the Berry curvature of solids using high-harmonic spectroscopy. *Nat. Commun.* **9**, 916 (2018).
6. Vampa, G. *et al.* All-Optical Reconstruction of Crystal Band Structure. *Phys. Rev. Lett.* **115**, 193603 (2015).
7. Lakhotia, H. *et al.* Laser picoscopy of valence electrons in solids. *Nature* **583**, 55–59 (2020).
